# Application of NGS molecular classification in the diagnosis of endometrial carcinoma: A supplement to traditional pathological diagnosis

**DOI:** 10.1002/cam4.5363

**Published:** 2022-11-07

**Authors:** Qunxian Rao, Jianwei Liao, Yangyang Li, Xin Zhang, Guocai Xu, Changbin Zhu, Shengya Tian, Qiuhong Chen, Hui Zhou, Bingzhong Zhang

**Affiliations:** ^1^ Department of Gynecologic Oncology, Sun Yat‐sen Memorial Hospital Sun Yat‐sen University Guangzhou People's Republic of China; ^2^ Cellular and Molecular Diagnostics Center, Sun Yat‐sen Memorial Hospital Sun Yat‐sen University Guangzhou China; ^3^ Department of Pathology, Sun Yat‐sen Memorial Hospital Sun Yat‐sen University Guangzhou China; ^4^ Department of Ultrasound, Sun Yat‐sen Memorial Hospital Sun Yat‐sen University Guangzhou China; ^5^ Department of Translational Medicine Amoy Diagnostics Co., Ltd. Xiamen China

**Keywords:** curettage, endometrial cancer, hysterectomy, molecular classification, NGS

## Abstract

**Objective:**

This study aims to demonstrate the advantages of NGS molecular classification in EC diagnosis and to assess whether molecular classification could be performed on curettage specimens and its concordance with subsequent hysterectomy specimens.

**Methods:**

80 patients with hysterectomy specimens and 35/80 patients with paired curettage specimens were stratified as *P*OLE mut, MSI‐H, *TP53* wt, or *TP53* abn group by NGS panel. Histotype, tumor grade, IHC results, and other pathological details were taken from original pathological reports.

**Results:**

The correlation analysis of 80 patients with hysterectomy specimens between NGS molecular classification and clinicopathological characters displayed that the *POLE* mut group was associated with EEC (87.5%) and *TP53* abn subtype was correlated to a later stage (Stage II–IV, 47.6%), G3 (76.2%), serous histology (61.9%) and myometrial invasion ≥50% (47.6%). A favorable concordance (31/32, 96.9%) was shown in MSI analysis and MMR IHC results, and the agreement rate of p53 IHC and *TP53* mutation was 81.5% (53/65). Compared with the p53 IHC abnormal group, the *TP53* mutation group had a higher correlation with high‐risk factors. A high level of concordance (31/35, 88.0%) of NGS molecular classification was achieved between curettage specimens and hysterectomy specimens while grade and histotype (including unclassified group) from curettage specimens and hysterectomy specimens showed only moderate levels of agreement, 54.3% (19/35) and 68.6% (24/35), respectively.

**Conclusion:**

NGS molecular classification achieved on curettage samples showed high concordance with the final hysterectomy specimens, demonstrating superior to the conventional pathological assessment of grade and histotype and potential utilization in clinical practice.

## Graphical abstract

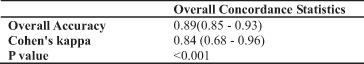

## 1 INTRODUCTION

Endometrial cancer (EC) is the most common gynecologic malignancy in China, with an increasing number of new diagnoses and a rapid mortality rate, which takes a heavy toll on women's health.^1^, ^2^, ^3^ The risk factors for EC include an excess of endogenous and exogenous estrogens, obesity, and others, and these factors can vary substantially between and within countries.^4^, ^5^ The gold standard treatment of EC is surgery, but for the patients with advanced disease, postoperative adjuvant treatment (chemotherapy, radiotherapy or targeted therapy) can be added to provide the best long‐term survival.^6^, ^7^, ^8^

The management of patients with EC largely relies on tumor histopathology. Traditional classification based on pathologic assessment includes Bokhman classification and WHO 2014 classification^9^, ^10^, ^11^ can be served as an important input for (adjuvant) treatment decisions, but has proved to be unreliable and has poor reproducibility. In addition, with considerable interobserver variation, high‐grade EC cannot be reliably classified according to histomorphologic criteria, resulting in misdiagnosis due to mixed high‐grade components in histology.^12^, ^13^, ^14^ Moreover, stage and lymphovascular space invasion (LVSI), parameters that are needed to define the risk group, are only available after definitive surgery,^15^, ^16^ such information comes too late for EC patients who may benefit from fertility‐sparing alternatives. This situation prompted research into developing biologically informative diagnostic tools to enhance the diagnosis and risk stratification of patients with EC.^17^ During the last decade, molecular characterization of EC is advancing rapidly. The Cancer Genome Atlas (TCGA) project for EC has shown how combinations of molecular features can be used to guide treatment decisions.^18^ According to different molecular profiles, TCGA divided EC into four distinct molecular subgroups: Polymerase‐epsilon (*POLE*) ultramutated, microsatellite instability hypermutated, a copy‐number low group with a low mutational burden, and a copy‐number high serous‐like group. Patients with *POLE*‐mutated ECs showed an excellent prognosis while the copy‐number high group have the worst.^19^ Unfortunately, the high cost of whole‐genome sequencing has greatly limited its practical application. For improvement, Talhouk A proposed a pragmatic molecular categorization model (ProMisE) based on *POLE* mutational analysis, mismatch repair protein immunohistochemistry (IHC), and p53 IHC.^20^ Though IHC is easier to carry out clinically, the interpretation of IHC is greatly affected by different expert pathologists, turning to unreliable conclusions. The emergence of next generation sequencing (NGS) has enabled the high comprehensive analysis of genomes and transcriptomes. Compared with Sanger sequencing, NGS has revolutionized the ability to sequence nucleic acids, and made genome sequencing more efficient and more cost‐effective.^21^ Jutta et al assessed molecular subtype using the ProMisE classifier and an NGS‐based approach, and compared the concordance between the two methods, the result showed excellent agreement.^22^ Together, these significant findings all point to indicating the potential of NGS use in clinical molecular subgrouping of EC, leaving it wide open for further exploration.

Assessment of preoperative tumor histologic subtype and grade using specimens taken from hysteroscopic biopsy, or dilatation and curettage is essential in determining the surgical staging.^23^ Studies revealed that there are limitations in preoperative sampling diagnosis because of inadequate tumor sampling or the observer variability, and the agreement rate for tumor grade between diagnostic sampling and post‐operative diagnosis varies from 32% to 97%.^24^, ^25^ Discordances may result in excessive or insufficient treatment, such as unnecessary surgical procedures like lymph node dissection. The emergence of molecular classification provides new insights into the shortcomings of traditional pathological diagnosis of diagnostic samples. The research illustrated that the concordance rate for pre‐ versus post‐surgery samples of TCGA classifier and ProMisE classifier are 92%^26^ and 89%,^27^ respectively, significantly higher than that of pathological diagnosis. At least to our knowledge, there is no documentation that NGS‐based testing can be performed on diagnostic endometrial specimens obtained prior to surgical staging and its concordance with subsequent hysterectomy specimens.

A simplified NGS panel is convenient for operation, easy to generalize, and with more accuracy, which might overcome the shortcomings of TCGA classifier and ProMisE classifier. On this basis, we applied a simplified NGS panel to a large population‐based cohort of Chinese EC patients to validate this tool, and additionally, compared discriminatory concordance between curettage and subsequent hysterectomy specimens.

## 2 METHODS

### 2.1 Patient cohort, sample collection, and pathological information collection

The study consists of a retrospective cohort of patients with EC from Sun Yat‐Sen Memorial Hospital, Sun Yat‐Sun University treated for EC between 2018 and 2021. Paraffin‐embedded (FFPE) tissue samples were selected from the biobank at the Department of Pathology. Of 120 patients, 40 patients were excluded owing to insufficient DNA quality. 35 of 80 patients with both curettage and hysterectomy endometrial specimens were picked out and used for further concordance analysis. The study was granted ethical approval SYSEC‐KY‐KS‐2021‐195.

### 2.2 MMR and P53 immunohistochemistry

MMR/P53 IHC staining was performed on full sections.^28^, ^29^ MMR were classified into two categories according to the MMR protein expression status: dMMR (protein expression of MLH1/PMS2/MSH2/MSH6 is negative) and MMR intact (all MMR proteins positively expressed).^28^ P53 was interpreted as abnormal if there was complete negative staining or strong/diffuse staining in 70% of tumor cells (aberrant expression pattern).^29^, ^30^

### 2.3 DNA extraction, library construction, and targeted sequencing

Genomic DNA (Tumor cell content ≥30%) from FFPE samples was extracted, purified, and quantified using MagPure FFPE DNA LQ Kit (Magen) according to regulated processes. For NGS analysis, a commercially available targeted AmoyDx EC Panel covering *POLE*, *TP53*, and MSI was used in this study (Amoy Diagnostics). DNA sequencing was performed on the NextSeq500 Illumina platform (Illumina). The average depth was 1000×, and the effective sequencing depth was greater than 300×. The proportion of Q30 bases was ≥75%. The sequencing data were analyzed by Sequencing Data Analysis Software (Amoy Diagnostics), the deleterious and suspected deleterious gene mutations were scrutinized and interpreted according to American College of Medical Genetics and Genomics (ACMG) guidelines and/or in ClinVar. The MSI phenotype detection method is based on the read count distribution of 55 specific microsatellite loci. A given threshold is set using the coverage ratio of a specific set of repeat lengths for each microsatellite locus, then the locus is categorized as unstable if the coverage ratio is less than the given threshold. MSS is categorized when the percentage of unstable loci is less than 15% in the given sample.

### 2.4 Statistical analysis

Histotype, tumor grade, IHC results, and other pathological details were taken from the original pathology report system in our center. The level of concordance for a molecular profile in both specimens was determined using overall accuracy and Cohen's kappa estimates. 95% confidence intervals are computed using the bootstrap approach with 1000 bootstrap samples.^31^

## 3 RESULTS

### 3.1 Patient cohort

**FIGURE 1:**
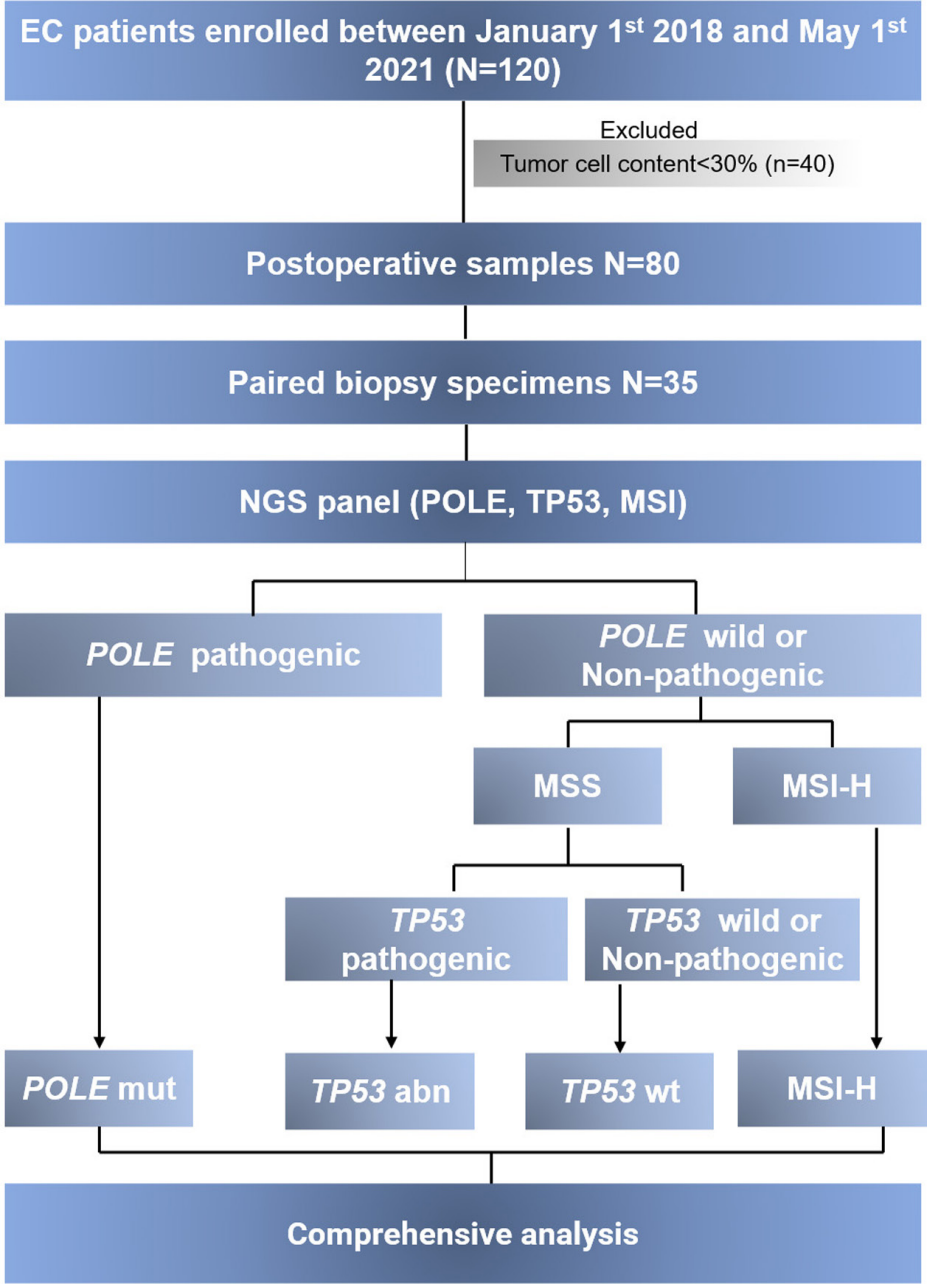
Study flow diagram. Abn, abnormal; MSI‐H, high microsatellite instability; MSS, microsatellite stable; No mut, no mutation; p53 nor, wild‐type p53; *POLE* mut, inactivating *POLE* exonuclease domain mutation; TP53 abn, null/missense p53 mutation.

The study flow chart is presented in Figure 1A. A total of 120 EC patients with post‐operative specimens from Sun Yat‐Sen Memorial Hospital between 2018 and 2021 were included. 40 patients were excluded for failed sequencing or insufficient tumor tissue for DNA extraction. The remaining 80 patients were compared for MMR/p53 IHC, grade, stages, histotype, and molecular subgroup assignment by a simplified NGS panel (Figure 1B). 35 of 80 cases that had paired curettage specimens were further analyzed of NGS‐based molecular classification and traditional pathological classification.

### 3.2 Description statistics of EC patients according to NGS molecular subgrouping

Eighty patients with post‐operative specimens were qualified for analysis. The pathological characteristics and demographics of these patients were described in Table 1. The median patient age at diagnosis was 60 years (range from 35 to 75 years). The majority of cases (62, 77.5%) were endometrioid histotypes, 4 (5%) were serous, and the rest were clear cell and mixed histology. Grade distribution shown 17 (21.3%) grade 1, 34 (42.5%) grade 2, and 29 (36.2%) grade 3.

**TABLE 1:** The descriptive statistics of patients according to NGS molecular subgroups as defined by post‐operative specimens

	Total	*POLE* mut	MSI‐H	*TP53* wt	*TP53* abn
Age at surgery					
Mean (SD)	56.0 (±8.0)	56.4 (±11.5)	53.5 (±3.9)	52.6 (±7.0)	61.2 (±8.3)
Median	55	58.0	53.0	51.0	64.0
Menopause					
Yes	52 (65.0%)	6 (75.0%)	11 (52.4%)	18 (60.0%)	17 (80.9%)
No	28 (35.0%)	2 (25.0%)	10 (47.6%)	12 (40.0%)	4 (19.1%)
Grade					
G1	17 (21.3%)	2 (25.0%)	6 (28.6%)	9 (30.0%)	0 (0.0%)
G2	34 (42.5%)	3 (37.5%)	9 (42.8%)	17 (56.7%)	5 (23.8%)
G3	29 (36.2%)	3 (37.5%)	6 (28.6%)	4 (13.3%)	16 (76.2%)
Histological subtype					
Endometrioid	62 (77.5%)	7 (87.5%)	18 (85.7%)	29 (96.7%)	8 (38.1%)
Serous	4 (5.0%)	0 (0.0%)	0 (0.0%)	0 (0.0%)	4 (19.0%)
Mixed*	13 (16.3%)	1 (12.5%)	3 (14.3%)	1 (3.3%)	8 (38.1%)
Clear cell	1 (1.2%)	0 (0.0%)	0 (0.0%)	0 (0.0%)	1 (4.8%)
Stage					
I	50 (62.5%)	5 (62.5%)	16 (76.1%)	18 (60.0%)	11 (52.4%)
II	6 (7.5%)	0 (0.0%)	1 (4.8%)	3 (10.0%)	2 (9.6%)
III	19 (23.8%)	3 (37.5%)	3 (14.3%)	9 (30.0%)	4 (19.0%)
IV	5 (6.2%)	0 (0.0%)	1 (4.8%)	0 (0.0%)	4 (19.0%)
LVSI					
Yes	33 (41.3%)	6 (75.0%)	9 (42.9%)	5 (16.7%)	13 (61.9%)
No	47 (58.7%)	2 (25.0%)	12 (57.1%)	25 (83.3%)	8 (38.1%)
Myometrial invasion					
None	7 (8.8%)	0 (0.0%)	2 (9.5%)	2 (6.7%)	3 (14.3%)
<50%	42 (52.5%)	5 (62.5%)	13 (61.9%)	16 (53.3%)	8 (38.1%)
≥50%	31 (38.7%)	3 (37.5%)	6 (28.6%)	12 (40.0%)	10 (47.6%)
Total	80 (100.0%)	8 (10.0%)	21 (26.2%)	30 (37.5%)	21 (26.3%)

*Note*: Mixed* includes mixed endometrioid with clear cell (3), mixed serous with endometrioid (9), and mixed endometrioid with large cell neuroendocrine carcinoma (1).

Abbreviations: Abn, abnormal; MSI‐H, high microsatellite instability; MSS, microsatellite stable; No mut, no mutation; p53 nor, wild‐type p53; *POLE* mut, inactivating *POLE* exonuclease domain mutation; TP53 abn, null/missense p53 mutation.

Molecular classification using simplified NGS panel yielded 4 molecular subgroups: 8 (10%) *POLE*, 21 (26.2%) MSI‐H, 30 (37.5%) *TP53* wt, and 21 (26.3%) *TP53* abn. A small proportion (5, 6.25%) of patients demonstrated more than one molecular feature. ECs harbored *POLE* exonuclease domain mutations were mostly endometrioid (87.5%) and consistent with previous findings, p53abn patients were older, serous (61.9%), higher rate of myometrial invasion ≥50% (47.6%) and were correlated with high stage and grade (G3 [76.2%], stages II ~ IV [47.6%]). The distribution of the four molecular subgroups with EC patients was similar to the proportion reported in previous studies (Figure S1), proving the accurate discriminatory ability of our NGS classifier.

### 3.3 Concordance between NGS‐based MSI status detection and MMR Immunohistochemistry on post‐operative specimens

Immunohistochemistry (the protein expression of MLH1, MSH2, MSH6, and PMS2) and pentaplex PCR‐based assays are the two common methodologies for the assessment of MSI phenotype in EC classification.^32^ In this study, we analyzed the MSI status using NGS based on read‐count distribution and compared the agreement with MMR IHC. A total of 32 cases with post‐operative specimens that had MMR (MLH1/PMS2, MSH2/MSH6) IHC reports in system from our center were picked out and subsequently performed NGS‐based MSI detection. Finally, 32 cases have been compared. As shown in Table S1, 25 cases were MMR IHC intact and their DNA sequencing results showed microsatellite stable (MSS), while 6 cases were MSI‐H and MMR protein‐deficient (Figure S2). However, one case with MSI‐H did not find expression loss for all four MMR genes. Thus, a high agreement rate (31/32, 96.9%) was observed between these two methods, inconsistency was only seen in one case.

### 3.4 Concordance of *TP53* mutational analysis and p53 Immunohistochemistry on post‐operative specimens

Scoring of p53 IHC sections between the three gynecologic pathologists was evaluated within the 65 hysterectomy samples. Complete negative staining or strongly positive (set to >70%, >80%, >85%, and > 90% of tumor cells for comparison in our study) was interpreted as abnormal. As detailed in Table S2, the concordance rate of p53 IHC and *TP53* mutational analysis lay between 60% to 81.5%, which was highest when using >80% and >85% of tumor cells as criteria, suggesting *TP53* mutational analysis could be utilized in helping p53 IHC define the right cutoff score of aberrant positive staining in clinical practice. Figure S3 showed consistency between expression patterns of p53 IHC and *TP53* mutation patterns. Information about 12 discordant cases were summarized in Table S3. Here we set an optimal threshold of tumor cell content ≥80%. Patients from 1 to 10 had high expression of *TP53* in protein level which was considered abnormal, while no DNA mutation was found. Conversely, both patient 11 and patient 12 detected *TP53* mutations.

To further explore the correlation between clinicopathological features and p53 IHC, and *TP53* mutation subgrouping, we analyzed the distribution of high‐risk features, including high grade, stage, LVSI, non‐endometrioid type, myometrial invasion (≥50%),^33^ in *TP53* abn and p53 IHC abn subgroup. Intriguingly, a much more common high‐risk features distribution was observed in *TP53* abn subgroup rather than p53 IHC abn subgroup (Table S4). *TP53* mutation can occur in *POLE* mut or MSI‐H EC, but is often considered a passenger mutation.^34^ When excluded EC with more than one molecular feature, the correlation between high‐risk features and TP53 abn subgroup turned out more significant.

### 3.5 Concordance of histotype and tumor grade between curettage and subsequent hysterectomy specimens

The descriptive analysis, including patient demographics, tumor grade, histotype, and molecular subgroups for 35 patients with paired curettage and subsequent hysterectomy specimens were detailed in Table 2 and Table S5. Table 3 showed the concordance metrics for grade and histotype. The overall concordance rate for grade and histotype was only 74.29% (26/35) and 54.29% (19/35), confirming the lack of reproducibility of the results of the pathologic assessments between curettage and hysterectomy specimens.^35^ Downgrading was found in 8.57% (3/35) and upgrading was found in 14.29% (5/35) of the cases, one representative discordant case was illustrated in Figure S4.

**TABLE 2:** The descriptive statistics of patients according to NGS molecular subgroups as defined by curettage specimen

	Total	*POLE* mut	MSI‐H	TP53 wt	TP53 abn
Age at surgery					
Mean (SD)	55.7 (±8.1)	51.4 (±10.5)	53.0 (±4.0)	54.9 (±6.8)	62.4 (±8.5)
Median	55.0	55.0	55.0	55.0	64.5
Menostasia					
Yes	24 (68.6%)	3 (60.0%)	5 (71.4%)	10 (66.7%)	6 (75.0%)
No	11 (31.3%)	2 (40.0%)	2 (28.6%)	5 (33.3%)	2 (25.0%)
Grade					
G1	6 (17.1%)	2 (40.0%)	1 (14.3%)	3 (20.0%)	0 (0.0%)
G2	10 (28.6%)	0 (0.0%)	3 (42.8%)	7 (46.7%)	0 (0.0%)
G3	10 (28.6%)	1 (20.0%)	1 (14.3%)	1 (6.7%)	7 (87.5%)
Unclassified	9 (25.7%)	2 (40.0%)	2 (28.6%)	4 (26.7%)	1 (12.5%)
Histological subtype					
Endometrioid	21 (60.0%)	3 (60.0%)	4 (57.1%)	11 (73.3%)	3 (37.5%)
Serous	1 (2.9%)	0 (0.0%)	0 (0.0%)	0 (0.0%)	1 (12.5%)
Mixed	2 (5.7%)	0 (0.0%)	0 (0.0%)	0 (11.1%)	2 (25.0%)
Clear cell	0 (0.0%)	0 (0.0%)	0 (0.0%)	0 (0.0%)	0 (0.0%)
Unclassified	11 (31.4%)	2 (40.0%)	3 (42.9%)	4 (26.7%)	2 (25.0%)
Total	35 (100.0%)	5 (14.3%)	7 (20.0%)	15 (42.9%)	8 (22.8%)

Abbreviations: MSI‐H, high microsatellite instability; *POLE* mut, inactivating *POLE* exonuclease domain mutation; TP53 abn, null/missense p53 mutation; TP53 wt, wild‐type p53.

**TABLE 3:** Concordance metrics of histotype and tumor grade between curettage and subsequent hysterectomy specimens

Curettage	Post‐operative					
	Endometrioid	Serous	Mixed	Clear cell	Unclassified	Total
Endometrioid	23 (65.7%)	0 (0%)	1 (2.9%)	0 (0%)	0 (0%)	24 (68.6%)
Serous	0 (0%)	1 (2.9%)	0 (0%)	0 (0%)	0 (0%)	1 (2.9%)
Mixed	0 (0%)	0 (0%)	2 (5.7%)	0 (0%)	0 (0%)	2 (5.7%)
Clear cell	0 (0%)	0 (0%)	0 (0%)	0 (0%)	0 (0%)	0 (0%)
Unclassified	6 (17.1%)	0 (0%)	2 (5.7%)	0 (0%)	0 (0%)	8 (22.8%)
Total	29 (82.8%)	1 (2.9%)	5 (14.3%)	0 (0%)	0 (0%)	35 (100%)

Curettage	Post‐operative samples				
	G1	G2	G3	Unclassified	Total
G1	3 (8.6%)	3 (8.6%)	0 (0%)	0 (0%)	6 (17.1%)
G2	1 (2.9%)	8 (22.8%)	1 (2.9%)	0 (0%)	10 (28.6%)
G3	0 (0%)	2 (5.7%)	8 (22.8%)	0 (0%)	10 (28.5%)
Unclassified	0 (0%)	3 (8.6%)	6 (17.1%)	0 (0%)	9 (25.7%)
Total	4 (11.5%)	16 (45.7%)	15 (42.8%)	0 (0%)	35 (100%)

*Note*: Neither unclassified EC histotype nor unclassified EC tumor grade was found.

### 3.6 Concordance of the molecular classification between curettage and subsequent hysterectomy specimens

Concordance metrics for NGS‐based molecular classification comparing curettage and subsequent hysterectomy specimens was illustrated in Table 4. The overall accuracy and Cohen's kappa value were 0.88 and 0.84, respectively (Table 5), demonstrating a great improvement over the conventional pathological assessment of grade and histotype.

**TABLE 4:** Concordance of the NGS molecular classification between curettage specimens and subsequent hysterectomy specimens

Curettage	Post‐operative samples				
	*POLE* mut	MSI‐H	TP53 wt	TP53 abn	Total
*POLE* mut	5 (14.3%)	1 (2.9%)	0 (0%)	0 (0%)	6 (17.1%)
MSI‐H	0 (0%)	6 (17.1%)	1 (2.9%)	0 (0%)	7 (20%)
TP53 wt	0 (0%)	0 (0%)	13 (37.1%)	1 (2.9%)	14 (40%)
TP53 abn	0 (0%)	0 (0%)	1 (2.9%)	7 (20%)	8 (22.9%)
Total	5 (14.3%)	7 (20%)	15 (42.9%)	8 (22.9%)	35 (100%)

Abbreviations: MSI‐H, high microsatellite instability; *POLE* mut, inactivating *POLE* exonuclease domain mutation; TP53 abn, null/missense p53 mutation; TP53 wt, wild‐type p53.

**TABLE 5:** Comparison of overall concordance statistics based on NGS molecular classification of curettage samples and post‐staging samples

	Overall Concordance Statistics
Overall Accuracy	0.89 (0.85–0.93)
Cohen's kappa	0.84 (0.68–0.96)
*p* value	<0.001

*Note*: 95% confidence intervals are computed using the bootstrap approach with 1000 bootstrap samples.

### 3.7 Interrogation of patients discordant on NGS molecular classification

Four patients (4/35) with discordant results between curettage and subsequent hysterectomy specimens as assessed by NGS classifier were summarized in Supplement table 6. In Cases 1, 3, and 4. The result was not changed after retesting and reassessment.

For case 1, a *POLE* mutation (p.T278A) was detected in the curettage sample but not in the post‐staging sample. The p.T278A is a missense mutation with uncertain clinical significance. The discordance might be related to the spatial heterogeneity of the tumor.

Case 2 showed discordant results in MSI status, with MSI‐H found in curettage but MSS in the final hysterectomy sample. Retesting of the sample using Sanger sequencing changed the curettage result to MSS.

Cases 3 and 4 showed discordancy in *TP53* mutation results and remained discordant after retesting. p.G245S and p.R175H are missense mutations. Reasons for discordance might also attribute to inadequate tumor sampling and spatial heterogeneity of the tumor.

## 4 DISCUSSION

Histological classification of EC provides important prognostic information to help determine appropriate surgery and adjuvant therapy.^23^ However, pathological classification by histological subtype and grade was considered highly subjective and has reproducibility challenges, and overlap between histological subtypes, and grade leads to complicating clinical decision‐making, and significant interobserver variability further hamper histological classification.^36^ Studies have shown interobserver disagreement is about 10%–20% and may reach 30% or higher in high‐grade endometrial carcinoma.^37^ Research from C Blake Gilks shows the agreement is only 62.5% for three pathologists.^12^ During the last decade, the EC molecular classification introduced by The Cancer Genome Atlas has provoked a transition toward molecular‐based classification with clear prognostic value. The TCGA project defined four distinct prognostic EC subtypes based on somatic copy number alterations (SCNA) and tumor mutational burden, raising the possibility of more precise guidance of adjuvant therapy, surgery, and disease surveillance.^35^ Although TCGA represented a meaningful step toward informative classification, it was impractical. Using a simplified molecular classifier that identifies four molecular subtypes that are analogous to TCGA, a highly successful rate (>95%) was shown in the PORTEC trials, demonstrating strong prognostic value in EC.^38^, ^39^ In this study, we applied a simplified NGS panel to categorize EC molecular type, and additionally, compared the level of concordance between endometrial biopsies and subsequent hysterectomy specimens.

The distribution of the four molecular subgroups identified by our NGS classifier was similar to the proportion reported in TCGA,^18^ demonstrating the discriminatory ability of our NGS classifier. Consistent with previous findings,^18^, ^40^, ^41^ p53 abn patients were older, mostly serous, and diagnosed at a high stage and grade (G3 [76.2%], stages II ~ IV [47.6%]). They showed other aggressive characteristics such as myometrial invasion (47.6%) and LVSI (61.9%). Notably, the subtype with the second most aggressive features was *POLE* in our study, with myometrial invasion (37.5%) and LVSI (75.0%) comparable to p53 abn tumors, and much higher than observation in women with MSI‐H or p53 wt ECs. The association with aggressive features of *POLE* subtype was also observed in other studies and was not related to their exceptionally better prognosis.^42^, ^43^ Taken together, our NGS panel‐based EC classification proved to be simplified and pragmatic, the result demonstrated the molecular feature of Chinese EC patients.

EC is a tumor type associated with MSI‐H and MMR.^44^ However, the result for immunohistochemical staining of MMR protein was not always consistent with DNA loci testing. In this study, we found one case with MSI‐H in DNA level but all four MMR protein expressions were intact. This may be caused by inactivating missense mutation which did not affect protein expression or inactivation of other repair genes such as *MSH3*. Immunohistochemical staining of tumors for p53 has a long history and has been widely used in clinical. Unfortunately, the interpretation of IHC results varies among observers, which complicates clinical decision‐making. Moreover, p53 proteins have relatively short half‐lives and their detection is therefore dependent on prompt fixation.^27^, ^45^ Here we attempted to establish an approach to provide auxiliary judgments for IHC results by correlating IHC patterns with the underlying mutation status, which pathologists can use in daily practice. It has been shown that the concordance between *TP53* mutation status and p53 overexpression, which was individually detected by NGS and immunohistochemistry, was 92% in EC.^30^ In gastric cancer, TP53 missense mutation, and p53 overexpression were highly consistent (90.9%).^46^ The concordance rate between p53 IHC and *TP53* mutation we observed reached the highest level at 81.5% when the IHC score standard was set at 80% of tumor cells, which suggests that *TP53* mutation status could be utilized in defining p53 IHC patterns. To further illustrate the correlation between clinicopathological features and p53 IHC, *TP53* mutation, we compared the proportion of high‐risk features in *TP53* abn subgroup and p53 IHC abn subgroup and intriguingly found that *TP53* abn group had an even higher proportion of high‐risk features than p53 IHC abn group, no matter *POLE* mut and dMMR tumors were excluded or not. One possible explanation of this finding is that not all p53 are uniformly expressed in all tissues. In pancreatic ductal adenocarcinoma, *TP53* missense mutation was confirmed to be associated with nuclear P53 overexpression in ≥25% of neoplastic cells.^47^ In diffuse large B‐cell lymphoma patients treated with R‐CHOP immunohistochemical analysis showing >50% cells expressing p53 protein was able to stratify patients with significantly different prognoses.^48^ The result also might be due to the smaller sample size in the study. Taken together, our results showed that NGS‐based *TP53* mutation analysis was highly consistent with IHC patterns, but has advantages in identifying high‐risk clinicopathological features, and could be utilized in defining IHC score.

Examination of curettage specimens is a common method for early diagnosis of EC and helps doctors obtain risk stratification in EC patients before operation.^49^ However, the histological types and grade of tumor differentiation of cancer diagnosed in endometrial curettage and post‐operative specimens were highly heterogenetic.^50^, ^51^ A study implicated that the highest concordance (127/148, 85.81%) between the histological diagnosis on curettage and post‐operative specimens were found for endometrioid carcinoma (21/44, 47.73%), and postoperative pathological examination is more accurate for diagnosis of atypical endometrial hyperplasia.^52^ In addition, in a study that compared the FIGO grades between preoperative endometrial samplings and hysterectomy specimens, the accuracy for grade 3 tumors (90.4%, *N* = 98) was significantly higher than for grade 1.^50^ In our research, the overall concordance rate of grade and histotype in curettage sample vs. final hysterectomy samples were 54.2% and 74.29%, with downgrading was found in 8.6% (3/35) and upgrading was found in 14.3% (5/35) of the endometrial samples. These data confirmed the previously reported lack of reproducibility when comparing curettage specimens with hysterectomy specimens. Unreliable histotype and grade assignment led to the inconsistent categorization of EC, which offered confusing information and brought barriers to clinical decision‐making. Conversely, reproducibility at a high level (88%) was seen with our NGS‐based molecular subclassification. Our study is the first to use NGS‐based molecular classification techniques for curettage specimens, and compare the molecular features of the curettage specimens with post‐operative specimens. In this way, we successfully classified all endometrial cancers using the NGS‐based classifier at initial diagnosis. Inconsistencies also arise, as in our study, which may be related to intratumor heterogeneity due to curettage of a small segment of tumor. As illustrated by Marta Brunetti,^53^ EC tumors are characterized by a high degree of tumoral heterogeneity.

Several limitations should be acknowledged in this study. Firstly, the overall sample size of our research was limited, particularly for those cases with curettage and paired hysterectomy specimens. Secondly, prognostic data are required to determine the prognostic discriminatory ability of the four molecular subgroups identified by our NGS classifier. Thirdly, the p53 IHC and MMR IHC results of curettage reports collected in our system were insufficient for statistics.

Molecular subgrouping has been fundamental in evolving the evaluation system and represents a new trend of EC, but there are still challenges that need to be solved. Using a small NGS panel, we have demonstrated the distribution of the four molecular subgroups in Chinese EC patients, and more importantly, be the first research that applied NGS method to curettage specimens and compared concordance between endometrial biopsies and subsequent hysterectomy specimens. We provided insight into the molecular classifier of EC, thus expanding its potential clinical application. Our next steps will focus on determining the optimal utilization of our NGS classifier in clinical trials, and explore whether our NGS classifier can guide fertility‐saving treatments, surgery, adjuvant therapy, and surveillance to improve outcomes for EC patients.

## AUTHOR CONTRIBUTIONS

**Qunxian Rao:** Writing – original draft (equal). **Jianwei Liao:** Writing – original draft (equal); writing – review and editing (equal). **Yangyang Li:** Data curation (equal). **Xin Zhang:** Data curation (equal). **Guocai Xu:** Methodology (equal). **Changbin Zhu:** Software (equal). **Shengya Tian:** Methodology (equal). **Qiuhong Chen:** Data curation (equal). **Hui Zhou:** Data curation (equal). **bingzhong zhang:** Conceptualization (equal); writing – original draft (equal).

## FUNDING INFORMATION

This study was supported by Grants from the National Natural Science Foundation of China (No. 81903043) and the Natural Science Foundation of Guangdong Province (No. 2018A030310086).

## CONFLICT OF INTEREST

Shengya Tian, Changbin Zhu, and Qiuhong Chen were employed by Amoy Diagnostics Co., Ltd. Other authors declared no conflict of interest.

## Supporting information


Figure S1

Figure S2

Figure S3

Figure S4

Table S1
Table S2
Table S3
Table S4
Table S5
Table S6

## Data Availability

Data sharing is not applicable to this article as no new data were created or analyzed in this study.
