# Supplementary figures and images for: Application of NGS molecular classification in the diagnosis of endometrial carcinoma: A supplement to traditional pathological diagnosis
Source: Cancer Med. 2022 Nov 7;12(5):5409–19. doi: 10.1002/cam4.5363 (PMC10028062; doi:10.1002/cam4.5363)

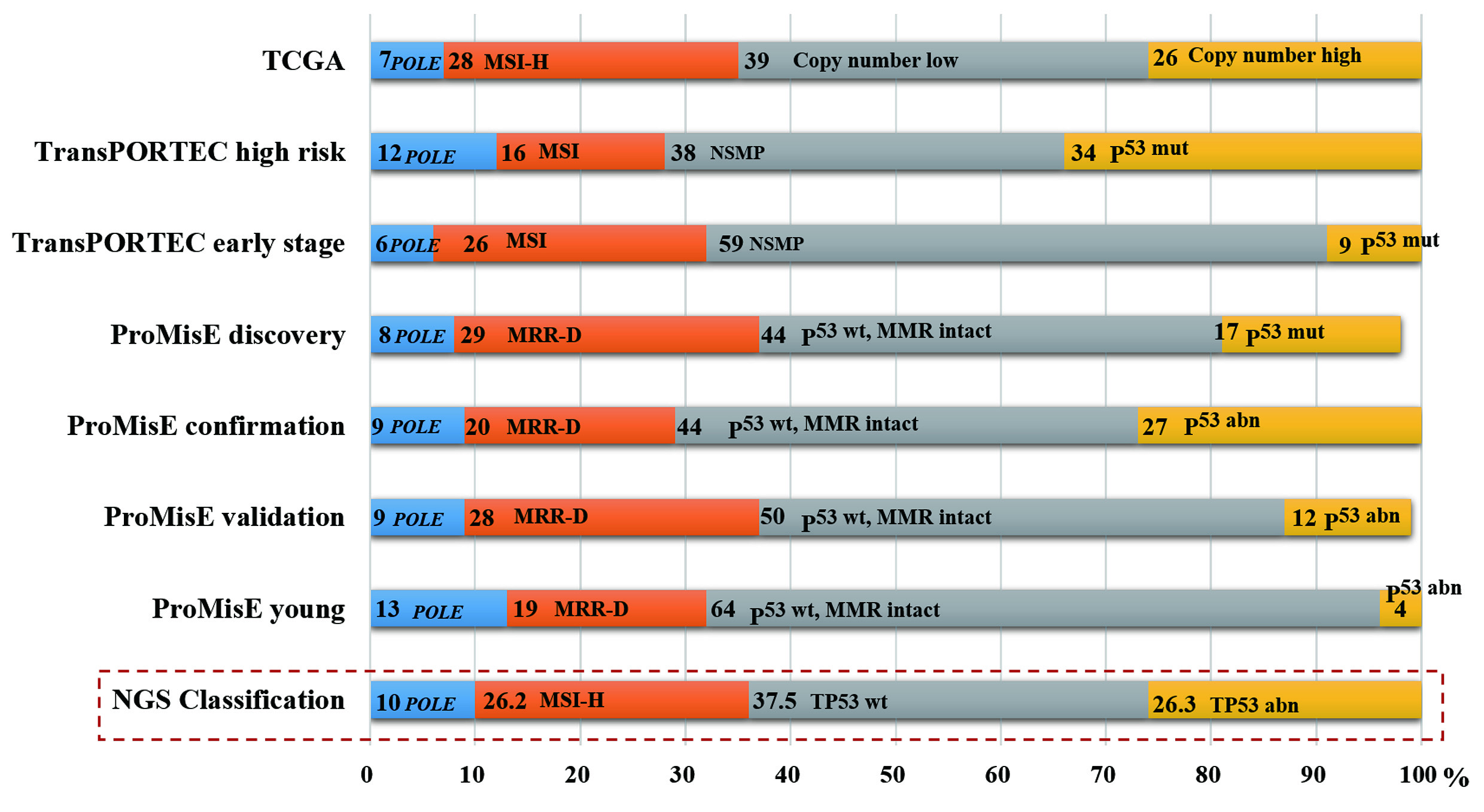

Supplement: Supplementary file 1 — Figure S1 [file CAM4-12-5409-s001.jpg]

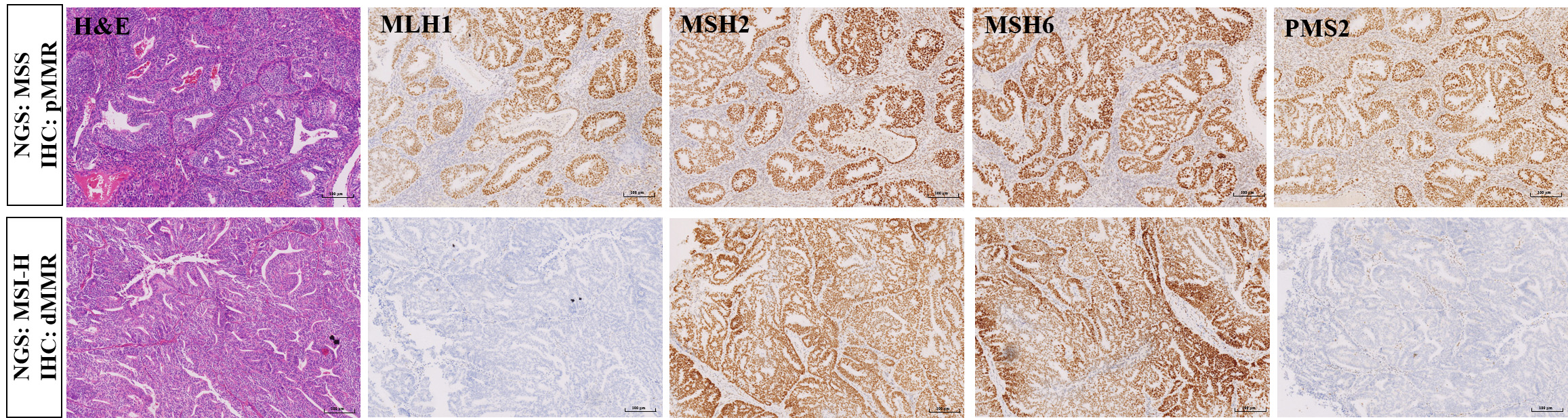

Supplement: Supplementary file 2 — Figure S2 [file CAM4-12-5409-s002.jpg]

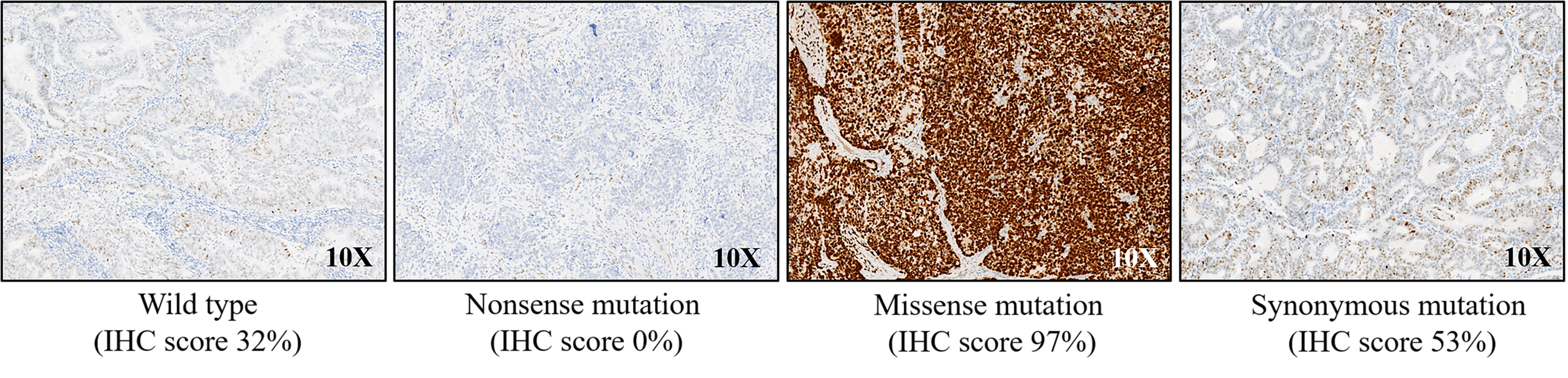

Supplement: Supplementary file 3 — Figure S3 [file CAM4-12-5409-s005.jpg]

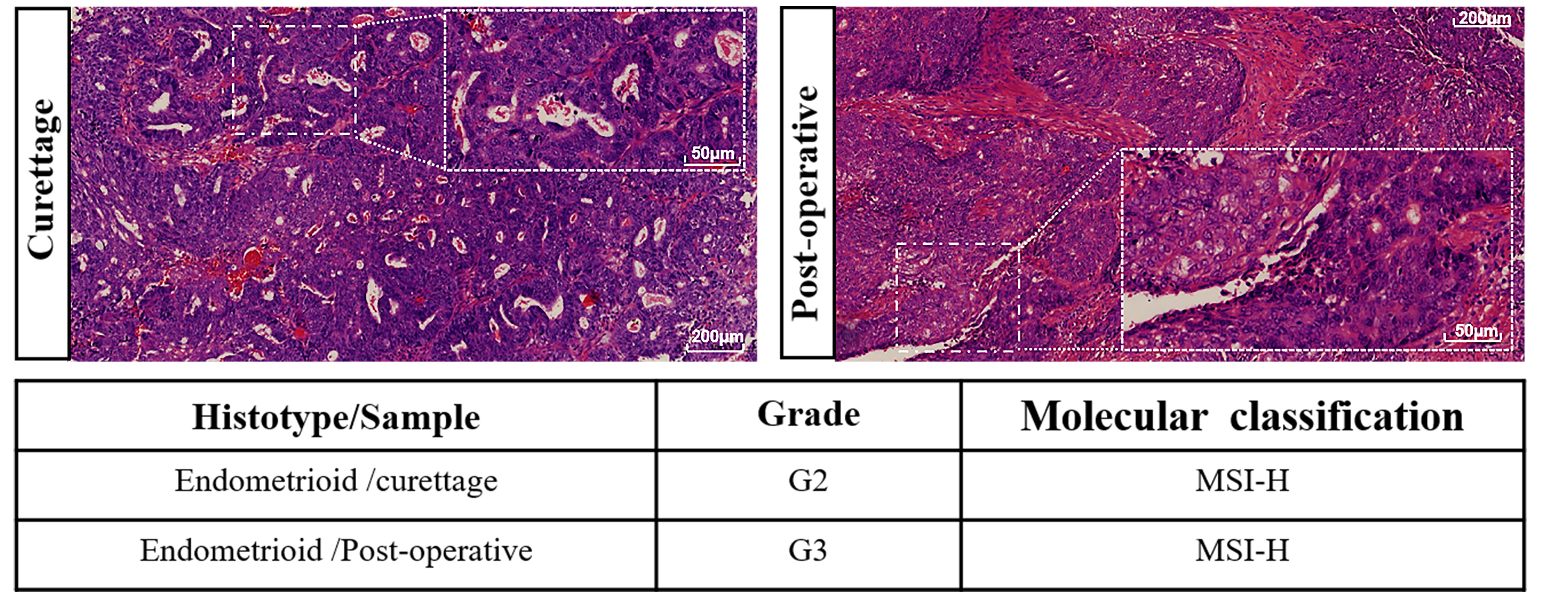

Supplement: Supplementary file 4 — Figure S4 [file CAM4-12-5409-s004.jpg]
