# Supplementary material for: Application of NGS molecular classification in the diagnosis of endometrial carcinoma: A supplement to traditional pathological diagnosis
Source: Cancer Med. 2022 Nov 7;12(5):5409–19. doi: 10.1002/cam4.5363 (PMC10028062; doi:10.1002/cam4.5363)
Supplement: Supplementary file 5 — Table S1 Table S2 Table S3 Table S4 Table S5 Table S6 [file CAM4-12-5409-s003.pdf]

Supplemental data

Table S1 Concordance between NGS-based MSI status detection and MMR Immunohistochemistry on post-operative specimens.

MSI status (NGS)	MMR IHC (MLH1、PMS2、MSH2、MSH6)		
	**pMMR**	**dMMR**	**Total**
**MSS**	25/32(78.1%)	0/32(0%)	25/32(78.1%)
**MSI-H**	1/32(3.1%)	6/32(18.8%)	7/32(21.9%)
**Total**	26/32(81.2%)	6/32(18.8%)	32/32(100%)

Abbreviations: pMMR=proficient mismatch repair; dMMR=deficient mismatch repair. Number in grey means consistency, number in red means discordance.

Table S2: Comparison of TP53 mutational analysis and p53 Immunohistochemistry in post-operative specimens.

TP53 Mutation	p53 IHC							
	**70%**		**80%**		**85%**		**90%**	
	**Nor**	**Abn**	**Nor**	**Abn**	**Nor**	**Abn**	**Nor**	**Abn**
**Wt**	26/65 (40.0%)	15/65 (23.1%)	10/65 (15.4%)	31/65 (47.8%)	7/65 (10.8%)	34/65 (52.3%)	5/65 (7.7%)	35/65 (53.8%)
**Abn**	24/65 (36.9%)	0/65 (0.0%)	22/65 (33.8%)	2/65 (3.1%)	19/65 (29.2%)	5/65 (7.7%)	16/65 (24.6%)	9/65 (13.9%)
**Agreement**	39/65 (60.0%)		53/65 (81.5%)		53/65 (81.5%)		51/65 (78.5%)	

Abbreviations: Wt=wild type; Nor=normal; Abn=abnormal. Through comparing TP53 DNA mutation and different IHC expression pattern (>70%, >80%, >85%, >90%), higher consistency was found only when p53 IHC positive rate reached to 80%.

Table S3: Cases shown discordance between TP53 mutational analysis and p53 IHC in post-operative specimens.

	**Patient**	**p53 IHC Staining (Staining ≥80% is overexpression)**			
		**pathologist A**	**pathologist B**	**Pathologist C**	**Average**
**p53 IHC abn & TP53 nor**	Patient1	90%	90%	90%	90%
	Patient2	90%	90%	90%	90%
	Patient3	70%	85%	90%	81%
	Patient4	90%	85%	90%	87%
	Patient5	95%	95%	90%	93%
	Patient6	90%	90%	90%	90%
	Patient7	95%	90%	80%	88%

For Review Only

	Patient8	85%	80%	85%	83%
	Patient9	90%	85%	85%	87%
	Patient10	80%	80%	85%	82%
**p53 IHC nor & TP53 abn**	Patient11	95%	75%	65%	78%
	Patient12	70%	70%	70%	70%

Abbreviations: p53 nor= wild-type p53; TP53 abn= null/missense p53 mutation. The positive rate of p53 IHC was firstly evaluated by 3 pathologists independently. Then we took the average as the final rate. The average positive rate (≥80%) will be considered p53 IHC abnormal.

Table S4: Distribution of high-risk pathological features in p53 IHC abn subgroup and TP53 abn subgroup.

	**G3**	**LVSI**	**Non-Endometrioid**	**Myometrial Invasion ≥50%**	**Stage Ⅱ-Ⅳ**
**TP53 abn**	20/28 (71.4%)	17/28 (60.7%)	13/28 (46.4%)	12/28 (42.9%)	11/28 (39.3%)
**p53 IHC abn**	17/32(53.1%)	17/32(53.1%)	14/32(43.8%)	10/32(31.2%)	12/32(37.5%)
**only TP53 abn**	16/21 (76.2%)	13/21 (61.9%)	13/21 (61.9%)	10/21 (47.6%)	10/21 (47.6%
**only p53 IHC abn**	15/24 (62.5%)	13/24 (54.2%)	12/24 (50.0%)	9/24 (37.5%)	11/24 (45.8%)

Abbreviations: G3=grade 3 endometrial cancer; LVSI=lymphovascular space invasion. Only Myometrial Invasion (≥50%) was accepted for calculating. Deep myometrial invasion (≥50%) is a prognostic factor for lymph node metastases and decreased survival in endometrial cancer.

Table S5: The descriptive statistics of patients according to NGS molecular subgroups as defined by paired post-operative samples. All percentages given are column percentages.

	**Total**	***POLE* mut**	**MSI-H**	**TP53 wt**	**TP53 abn**
**Age at Surgery**					
Mean (SD)	55.7(±8.1)	51.4(±10.5)	53.0(±4.0)	54.9(±6.8)	62.4(±8.5)
Median	55.0	55.0	55.0	55.0	64.5
**Menostasia**					
Yes	24(68.6%)	3(60.0%)	5(71.4%)	10(66.7%)	6(75.0%)
No	11(31.4%)	2(40.0%)	2(28.6%)	5(33.3%)	2(25.0%)
**Grade**					
G1	4(11.4%)	1(20.0%)	1(14.4%)	2(13.3%)	0(0.0%)
G2	16(45.7%)	1(20.0%)	3(42.8%)	11(73.3%)	1(12.5%)
G3	15(42.9%)	3(60.0%)	3(42.8%)	2(13.3%)	7(87.5%)
**Histological Subtype**					
Endometrioid	29(82.9%)	4(80.0%)	7(100.0%)	15(100.0%)	3(37.5%)
Serous	1(2.8%)	0(0.0%)	0(0.0%)	0(0.0%)	1(12.5%)
Mixed	5(14.3%)	1(20.0%)	0(0.0%)	0(10.0%)	4(50.0%)
Clear cell	0(0.0%)	0(0.0%)	0(0.0%)	0(0.0%)	0(0.0%)
Unclassified	0(0.0%)	0(0.0%)	0(0.0%)	0(0.0%)	0(0.0%)
**Stage**					

For Review Only

Ⅰ	20(57.1%)	2(40.0%)	5(71.4%)	7(46.7%)	6(75.0%)
Ⅱ	4(11.5%)	0(0.0%)	1(14.3%)	3(20.0%)	0(0.0%)
Ⅲ	11(31.4%)	3(60.0%)	1(14.3%)	5(33.3%)	2(25.0%)
**LVSI**					
Yes	13(37.1%)	4(80.0%)	3(42.9%)	1(6.7%)	5(62.5%)
No	22(62.9%)	1(20.0%)	4(57.1%)	14(93.3%)	3(37.5%)
**Myometrial Invasion**					
None	2(5.7%)	0(0.0%)	0(0.0%)	1(6.7%)	1(12.5%)
＜50%	18(51.4%)	3(60.0%)	5(71.4%)	6(40.0%)	4(50.0%)
≥50%	15(42.9%)	2(40.0%)	2(28.6%)	8(53.3%)	3(37.5%)
**Total**	35(100.0%)	5(14.3%)	7(20.0%)	15(42.9%)	8(22.8%)

Abbreviations: *POLE* mut=inactivating *POLE* exonuclease domain mutation; TP53 Wt= wild-type p53; TP53 Abn= null/missense p53 mutation. LVSI=lymphovascular space invasion.

For Review Only

Table S6: Interrogation of cases discordant on NGS molecular classification between curettage and subsequent hysterectomy specimens.

Cases	Specimen	MSI Status	*POLE*	TP53	Molecular type	Retesting	Final type
1	curettage	MSI-H	p.T278A	R175H	*POLE* mut	Retest validates	*POLE* mut
	resection	MSI-H	no mut	R213Q, R175H	MSI-H	No *POLE* mut	MSI-H
2	curettage	MSI-H	no mut	wt	MSI-H	Changed to MSS	TP53 wt
	resection	MSS	no mut	wt	TP53 wt	Confirms MSS	TP53 wt
3	curettage	MSS	no mut	wt	TP53 wt	Retest validates	TP53 wt
	resection	MSS	no mut	p.(G245S)	TP53 abn	Retest validates	TP53 abn
4	curettage	MSS	no mut	p.(R175H)	TP53 abn	Retest validates	TP53 abn
	resection	MSS	no mut	wt	TP53 wt	Retest validates	TP53 wt

Abbreviations: MSI-H=high microsatellite instability; MSS=microsatellite stable; Abn=abnormal. No mut=no mutation; TP53 wt= wild-type p53; TP53 abn= null/missense p53 mutation. 4 cases with both curettage and resection samples were retested and final type as shown in the last column.
